# Generalized lymphadenopathy in the presence of acute Epstein–Barr virus infection as the initial manifestation of systemic lupus erythematous: A case report

**DOI:** 10.1002/ccr3.9134

**Published:** 2024-07-01

**Authors:** Kimia Jazi, Zahra Faraji, Fateme Aghaei, Alireza Shahhamzeh, Reihane Tabaraii, Maryam Masoumi

**Affiliations:** ^1^ Student Research Committee Faculty of Medicine, Medical University of Qom Qom Iran; ^2^ Clinical Research and Development Center Shahid Beheshti Hospital, Qom University of Medical Sciences Qom Iran; ^3^ School of Medicine Shahid Beheshti Hospital, Qom University of Medical Sciences Qom Iran

**Keywords:** case report, EBV, lymphadenopathy, systemic lupus erythematous

## Abstract

**Key Clinical Message:**

Clinicians should carefully consider generalized lymphadenopathy, particularly post viral infections, as one of the possible systemic lupus erythematous (SLE) first signs regarding unusual joint involvements such as sacroiliitis. Late diagnosis of this autoimmune inflammatory disease, could lead to irreversible morbidity and higher mortality.

**Abstract:**

Lymphadenopathy could represent various etiologies, including infections, malignancies, and rheumatologic diseases. SLE is known as the great mimicker which could be presented with different first manifestations. We report a 42‐year‐old woman in the acute phase of Epstein–Barr infection, admitted with polyarticular peripheral arthritis, sacroiliitis, and generalized lymphadenopathy. She had no similar history or taken unpasteurized dairy. Nodes were soft, mobile, and tender without skin change on top. During the process, she was diagnosed with SLE and discharged with prednisolone 30 mg/day and hydroxychloroquine 400 mg/day. After 2 weeks of follow‐up, all lymphadenopathy and symptoms were diminished. This case underscores the thousand faces innate of SLE. Clinical awareness would lead to an accurate diagnosis and early intervention.

## 1 INTRODUCTION

Systemic lupus erythematous (SLE) is a complex heterogenous disease with variable clinical presentations.^1^ The prevalence of SLE was reported 40/100,000 among Iranian population.^2^ Pathogens have proposed to cause autoimmunity. Epstein–Barr virus (EBV), since 1969, has been frequently associated with SLE. Acute inflammation and the production of auto‐antibodies in individuals with predisposing genetic and environmental factors could lead to a chronic inflammatory state such as SLE disease.^3^ SLE is a multifaceted and innovative disease that can have catastrophic impacts on any organ system. A typical SLE patient could present with multiple symptoms from any organ system. Fever, myalgias, fatigue, weight loss, arthralgia, and lupus nephritis are the most commonly presented manifestations. Less frequently, patients can also present with neuropsychiatric manifestations, myocardial infarctions, thromboembolic diseases and vasculitis. According to its wide variety of manifestations, SLE is known as the “great mimicker.” There has been reports concerning atypical manifestations such as atypical chest pain and elevated troponin levels concerning for acute coronary syndrome, acute cutaneous LE, bullous LE, and enteritis and cystitis.^4^, ^5^

Herein we report 42‐year‐old women presented with generalized lymphadenopathy and fever in the presence of EBV infection, as initial manifestations of SLE followed by sacroiliitis for the first time.

## 2 CASE PRESENTATION

### 2.1 Case history/Examination

A 42‐year‐old woman was admitted to our hospital with myalgia, arthralgia, gait impairment, lower limb paresthesia, and pain in the left hip radiating to the anterior compartment of the thigh for 2 months. The pain would lessen with heat and exacerbated with activity. She also mentioned a 2 months history of night sweat but no weight loss. No history of aphthous or any similar mucocutaneous lesions was recorded, neither she had eating raw or unpasteurized dairy products. Her past medical history was remarkable for diabetes mellitus, ischemic heart disease, and hypothyroidism. The patient was afebrile with stable vital signs. In the physical examination, she had generalized lymphadenopathy involving inguinal, axillary, and cervical nodes on both sides with approximately 2 × 1 cm, 2 × 2 cm, and 1.5 × 2 cm, respectively. Nodes were soft, mobile, and painful with no skin changes on top.

## 3 METHODS

### 3.1 Differential diagnosis, investigations and treatment

Laboratory test results on admission date were as follows: WBC: white blood cell (WBC), 16,600/μL (4000–11,000) with shift to left (71.2% neutrophil); hemoglobin (Hb), 13 g/dL (12–16); platelets (plts), 16.2 × 104/μL (15–45 × 104); C‐reactive protein (CRP), 56.2 mg/dL (positive >9, negative <6); erythrocyte sedimentation rate (ESR), 67; creatinine (Cr), 1 mg/dL (0.6–1.2); blood sugar (BS), 79 mg/dL; lactate dehydrogenase (LDH), 478 U/L; long with normal liver function tests and electrolytes (Table 1).

**TABLE 1:** Initial laboratory tests of the patient results.

Test, Unit	Result	Reference range
WBC, μL	16,600	4000–11,000
RBC, ×10^6^ μL	4.33	4.2–6.3
Hb, g/dL	13	12–16
Platelet, μL	162,000	150,000–450,000
MCV, fL	86.1	80–100
Neutrophil, %	71.2%	—
CRP, mg/dL	56.2	Negative: <6, Positive: >9
ESR 1 h, mm/hr	67	—
BS, mg/dL	79	70–120
Urea, mg/dL	34	10–50
Creatinine, mg/dL	1	0.6–1.2
AST, U/L	21	Up to 35
ALT, U/L	16	Up to 45
ALP, U/L	109	98–279
LDH, U/L	622	225–500
CPK Total, U/L	19	—
Aldolase, U/L	26.5	<7.6
Uric Acid, mg/dL	3.1	Male 3.4–7, Female 2.4–5.7
Na, mmol/L	141	135–148
K, mmol/L	4.3	3.5–5.3

Abbreviations: ALP, alkaline phosphatase; ALT, alanine transaminase; AST, aspartate aminotransferase; BS, blood sugar; CPK, creatinine phosphokinase; CRP, c‐reactive protein; ESR, erythrocyte sedimentation rate; Hb, hemoglobin; K, potassium; LDH, lactate dehydrogenase; MCV, mean corpuscular volume; Na, sodium; RBC, red blood cell; WBC, white blood cell.

Sonographic assessments showed several prominent lymph nodes with 15 × 20 mm, 15 × 22 mm, 19 × 11 mm, and 17 × 20 mm in paraaortic, porta hepatis, inguinal, and left iliac in addition to mild splenomegaly. The chest computed tomography (CT) scan showed normal lung parenchyma; however, a prominent axillary lymph node about 2 cm long was available. No supraclavicular or mediastinal lymph node was detected. Sputum smear analysis was normal. Following, acid fast bacilli was not seen and the bronchoscopy evaluation was negative for any pathological finding. At first, infectious causes were ruled out. Tests for brucellosis were all negative as well as Hepatitis B antibody (HBs Ab); coronavirus disease of 2019 (Covid‐19) real time polymerase chain reaction (RT‐PCR); Human Immunodeficiency Virus antibody (HIV Ab); Cytomegalovirus immunoglobulin G (CMV IgG), 47.8 (positive > = 22); CMV IgM, 0.31 (positive > = 0.9); Epstein–Bar Virus Viral Capsid Antigen IgG (EBV‐VCA), 0.4 (positive > = 1); and EBV IgM, 4 (positive >1.1) (Table 2).

**TABLE 2:** Specific immunologic tests of the patient.

Test, Unit	Result	Reference Range
2‐ME	Negative	—
Indirect Coombs	Negative	—
Wright Agglutination Test	Negative	—
COVID‐19 RT‐PCR	Negative	—
HBs Ab	Negative	—
HIV Ab	Negative	—
CMV IgG	47.8	Positive > = 22
CMV IgM	0.31	Positive > = 0.9
EBV‐VCA IgG	0.4	Positive > = 1
EBV‐VCA IgM	4	Positive >1.1
CA‐125, U/mL	32.5	Negative = <35
CA 19–9, U/mL	34.10	Negative <40
AFP, ng/mL	0.5	0.2–8.5
CEA, ng/mL	1.20	0.3–5
FANA	1:100	Positive reaction at 1:100 or more
Anti‐ds DNA AB, IU/mL	46.21	Positive >18
C3, mg/dL	68	90–160
C4, mg/dL	5.9	10–40
CH50, %	92	41.2–95
Anti B2‐GLP1 antibody (IgG)	5	Positive: >20
Anti B2‐GLP1 antibody (IgM)	4	Positive: >20
ACA IgG	9	Positive: > = 12
ACA IgM	1.9	Positive: > = 12
LA antibody (dRVVT)	33	Direct: 25–45
LA antibody (aPTT)	29	After mixing: 25–45
Anti‐CCP AB	17.5	Positive >18
RF	Negative	—
Anti‐SSA, RU/mL		Positive >18
Anti‐SSB, RU/mL		Positive >18

Abbreviations: 2ME, 2‐Mercaptoethanol; ACA, anti‐cardiolipin antibody; AFP, Alpha‐fetoprotein; Anti B2‐GLP1 antibody, anti‐b2glycoprotein antibody; anti‐CCP Ab, Anti‐cyclic citrullinated peptide antibody; Anti‐ds DNA AB, anti‐double stranded DNA antibody; anti‐SSA and B, Sjögren's‐syndrome‐related antigen A and B; aPPT, activated partial prothrombin time; C3 and C4, complement 3 and 4; CA, Cancer Antigen; CEA, carcinoembryonic antigen; CH50, a total hemolytic complement; CMV IgG and IgM, Cytomegalovirus immunoglobulin G and M; dRVVT, Diluted Russell Viper Venom Time; EBV‐VCA, Epstein Bar Virus Viral Capsid Antigen; FANA, fluorescent antinuclear antibody; HBs Ab, Hepatitis B antibody; HIV Ab, Human Immunodeficiency Virus antibody; LA antibody, lupus anticoagulant; RF, Rheumatoid Factor.

These findings led to the possible diagnosis of lymphoma. Peripheral blood smear showed hypereosinophilia. Whole body bone scan was negative for any bone metastasis. However, excision of inguinal lymph nodes showed reactive lymph node with follicular hyperplasia and absence of malignancy or granuloma. Laboratory test results after 14 days of admission were as follows: ESR, 102 mm/h; CRP, 18.4; cancer antigen (CA)‐125, 32.5 U/mL (= < 35); CA 19–9, 34.10 U/mL (<40); Alpha‐fetoprotein (AFP), 0.5 ng/mL (0.2–8.5); carcinoembryonic antigen (CEA), 1.20 ng/mL (0.3–5). Bone marrow aspiration reported the absence of malignancy. Lymphoma excluded, thus, in the third place; rheumatologic diseases were considered. No alopecia, rash, oral ulcers, photosensitivity, neurological disorder, or bladder irritation was noted. In the second physical examination, we found general inflammatory polyarthritis of small joints of hand, shoulder, and elbow. Magnetic resonance imaging (MRI) of lumbosacral joint revealed slight disc bulging at L_4_‐L_5_ and L_5_‐S_1_ joints. Hip MRI showed was normal and sacroiliac imaging highlighted mild sacroiliitis with left dominance without subchondral erosion. Laboratory test results were as follows fluorescent antinuclear antibody (FANA) (indirect immunofluorescence test) 1:100 (normal<1: 100) cytoplasmic and nucleoplasm granular; a total hemolytic complement (CH_50_), 92% (41.2–95); complement 3 (C_3_), 68 mg/dL (90–160); C_4_, 5.9 mg/dL (10–40); anti‐double stranded DNA antibody (anti‐ds DNA), 46.21 IU/mL (positive >18); anti‐cyclic citrullinated peptide antibody (anti‐CCP Ab), 17.5 (positive >18); Rheumatoid Factor (RF), negative; Sjögren's‐syndrome‐related antigen A (anti‐SSA), 6.77 RU/ml (positive >18); anti‐SSB, 9.43 RU/ml (positive >18) (Table 2).

## 4 RESULTS

### 4.1 Outcome and follow‐up

The patient fulfilled European League Against Rheumatism (EULAR) and American College of Rheumatology (ACR) 2019 criteria for SLE with 16 points (FANA = 1:100, inflammatory polyarthritis (6 points), as well as low C_3_ and C_4_ (4 points), and increased anti‐ds DNA (6 points)).^6^ Intravenous administration of methylprednisolone (1000 mg/day) was started immediately and continued for 3 days. Finally, after 12 days she was discharged with prednisolone 30 mg/day (0.5 mg/kg/day) and hydroxychloroquine 400 mg/day. After 2 weeks of follow‐up, all lymphadenopathy and symptoms were diminished.

Regarding her poor compliance, she did not check for her follow‐up sessions, hesitated taking medications and a year later, she was expired due to severe neuropsychiatric SLE with fever and cerebritis in another hospital.

## 5 CASE DISCUSSION

Lymphadenopathy constitutes a vast majority of etiologies including infections (bacterial, brucellosis, tuberculosis; viral, HIV, EBV, herpes simplex virus, CMV, hepatitis B), cancer (lymphoma, leukemia), sarcoidosis, lupus erythematosus, amyloidosis, rheumatoid arthritis, and etc. To outline the cause of lymphadenopathy, a history, physical examination, and laboratory tests are obtained.

Although the exact gene–environment interactions remain vague, SLE includes multiple immunologic components such as hyperactivation of B cells, T cells, and monocytes resulting in the production of countless antibodies, autoantibodies, and cytokines.^5^ The clinical presentation and evolution of SLE consider an extensive variety. The 2019 EULAR/ACR released the most recent classification criteria for SLE.^6^ The criteria have two separate parts; clinical and immunological features. Clinical involvements consider constitutional, hematological, neuropsychiatric, mucocutaneous, serosal, musculoskeletal, and renal system and immunological criteria include the presence of ANA, antiphospholipid antibodies, complement proteins and SLE‐specific antibodies like anti‐ds DNA, anti‐Smith, and anti‐histone DNA.^6^ To meet the criteria, the patient must represent at least one clinical criterion, positive ANA along with more than 10 points.

Lupus lymphadenopathy has been reported in 5%–7% in the newly diagnosed patients.^7^ It has been found that SLE patients first presented with lymphadenopathy more probably show constitutional symptoms such as fever, fatigue, and weight loss, hepatomegaly and splenomegaly, and additionally, decreased complements along with increased anti‐dsDNA indicating that lymphadenopathy is a sign of disease activity.^6^ In the patient currently reported, the diagnosis of SLE was followed by ruling out lymphoma, infections and other causes of lymphadenopathy. Our patient met the criteria with 16 points, presenting with generalized lymphadenopathy as the first manifestation.

Interestingly, EBV VCA IgM results of the 42‐year‐old patient was positive representing an acute EBV infection. To date, there has been multiple studies approving the association of SLE flare and EBV infection similar to our patient.^8^, ^9^ In a cross‐sectional study of 40 patients with SLE, the EBV test reported positive in 67.5%, while half of patients had active disease.^10^ The viral load was significantly higher in patients with active disease independent of immunosuppressive medication. Another study also showed that SLE patients with positive EBV‐VCA IgA had a higher prevalence of disease flare, confirming the association between EBV reactivation and SLE flare.^11^ EBV targets naïve B cells, by germinal center reaction, the infected cells enter the memory B cell group, consequently, the virus maintain the latency. Noncoding RNAs expressed by EBV, regulate B cell survival and induce the secretion of interferons by plasmacytoid dendritic cells, which cause unregulated T cell activation which could mimic SLE.^3^ Moreover, Immunoglobin (Ig) A deficiency found in 6% of SLE patients could also counteract an epithelial EBV reactivation.^12^ Another mechanism contribute to molecular mimicry; Epstein–Barr virus nuclear antigen 1 (EBNA‐1) has shown cross reactions with SLE‐related autoantigens, resulting in the development of SLE.^13^ Our patient had could be one of many SLE patients with positive EBV, presenting with disease activation by any of the mentioned mechanisms, however, this case is the first report of atypical presentation of SLE with positive acute serological EBV. We suggest further large‐scale studies to evaluate the association of EBV and frequency of SLE activation, the clinical presentations, and prognosis. Furthermore, we suggest that positive EBV test could be possibly a sign for early diagnosis in patients with potential risk factors who do not complete SLE diagnosis criteria. This should be evaluated in long‐term large‐scale observations.

Additionally, she had symptoms, signs, and an MRI report of mild sacroiliitis which is scarcely reported in SLE patients.^9^ Recently, a study represented higher titer of CRP as a potential risk factor of sacroiliac involvements in lupus patients.^14^ We suggest that other risk factors such as EBV reactivation, presence of different lupus specific antibodies should also be evaluated to predict possibility of sacroiliitis as well as rare first manifestations.

## 6 CONCLUSION

Lymphadenopathy is considered one of rare onset manifestations. There have been previous case reports of lupus lymphadenopathy; however, the correlation between these nonfrequent presentations and viral reactivations remain vague. Future investigations should be performed to clarify possible risk factors of rare involvements of SLE in order to prevent a late diagnosis. More importantly, physicians should consider SLE as one of the prior differential diagnoses of lymphadenopathy for which a proper history, physical and laboratory examination, and lymph node biopsy is needed.

## AUTHOR CONTRIBUTIONS

**Kimia Jazi:** Validation; writing – review and editing. **Zahra Faraji:** Writing – original draft. **Fateme Aghaei:** Writing – original draft. **Alireza Shahhamzeh:** Writing – review and editing. **Reihane Tabaraii:** Writing – original draft. **Maryam Masoumi:** Writing – review and editing.

## FUNDING INFORMATION

None.

## CONFLICT OF INTEREST STATEMENT

The authors declare no competing interests.

## CONSENT

Written informed consent was obtained from the patient to publish this report in accordance with the journal's patient consent policy.

## Data Availability

All data generated or analyzed during this study are included in this published article.
